# On-Site Blackwater Treatment Fosters Microbial Groups and Functions to Efficiently and Robustly Recover Carbon and Nutrients

**DOI:** 10.3390/microorganisms9010075

**Published:** 2020-12-30

**Authors:** Eiko E. Kuramae, Mauricio R. Dimitrov, Gustavo H. R. da Silva, Adriano R. Lucheta, Lucas W. Mendes, Ronildson L. Luz, Louise E. M. Vet, Tania V. Fernandes

**Affiliations:** 1 Department of Microbial Ecology, Netherlands Institute of Ecology (NIOO-KNAW), Droevendaalsesteeg 10, 6708 PB Wageningen, The Netherlands; mau_dimitrov@hotmail.com (M.R.D.); arlucheta@gmail.com (A.R.L.); l.mendes@nioo.knaw.nl (L.W.M.); luzrnd@gmail.com (R.L.L.); 2 Ecology and Biodiversity, Institute of Environmental Biology, Utrecht University, Padualaan 8, 3584 CH Utrecht, The Netherlands; 3 Department of Environmental and Civil Engineering, São Paulo State University (UNESP), Bauru 17033-360, Brazil; gustavo.ribeiro@unesp.br; 4 Department of Terrestrial Ecology, Netherlands Institute of Ecology (NIOO-KNAW), Droevendaalsesteeg 10, 6708 PB Wageningen, The Netherlands; l.vet@nioo.knaw.nl; 5 Department of Aquatic Ecology, Netherlands Institute of Ecology (NIOO-KNAW), Droevendaalsesteeg 10, 6708 PB Wageningen, The Netherlands; T.Fernandes@nioo.knaw.nl

**Keywords:** blackwater microbiome, phosphorus, nitrogen, *Chlorella sorokiniana*, shotgun metagenomics

## Abstract

Wastewater is considered a renewable resource water and energy. An advantage of decentralized sanitation systems is the separation of the blackwater (BW) stream, contaminated with human pathogens, from the remaining household water. However, the composition and functions of the microbial community in BW are not known. In this study, we used shotgun metagenomics to assess the dynamics of microbial community structure and function throughout a new BW anaerobic digestion system installed at The Netherlands Institute of Ecology. Samples from the influent (BW), primary effluent (anaerobic digested BW), sludge and final effluent of the pilot upflow anaerobic sludge blanket (UASB) reactor and microalgae pilot tubular photobioreactor (PBR) were analyzed. Our results showed a decrease in microbial richness and diversity followed by a decrease in functional complexity and co-occurrence along the different modules of the bioreactor. The microbial diversity and function decrease were reflected both changes in substrate composition and wash conditions. Our wastewater treatment system also decreased microbial functions related to pathogenesis. In summary, the new sanitation system studied here fosters microbial groups and functions that allow the system to efficiently and robustly recover carbon and nutrients while reducing pathogenic groups, ultimately generating a final effluent safe for discharge and reuse.

## 1. Introduction

The global demand for freshwater is rising at a rate of approximately 1% per year due to population growth, changes in consumption patterns, and economic development [1]. This increased demand comes as freshwater availability is decreasing due to climate change, unsustainable ground water consumption and other human activities [2]. Due to this unsustainable impact on the global water cycle, a growing number of people are living under conditions of water scarcity and reduced water quality, endangering aquatic ecosystems and public health [1]. Poor surface water and groundwater quality are due largely to a lack of wastewater treatment. According to a recent UN report, 80% of all wastewater (domestic and industrial) is discharged without treatment, resulting in physical, chemical and biological pollution [1]. Consequently, improving sanitation by increasing access to wastewater treatment is a priority in the UN’s Sustainable Development Goals (SDG 6) as a recognized human right that is essential for food security, health promotion and poverty reduction [3,4]. Wastewater treatment can be applied in a centralized or decentralized manner. While the former requires a sewer connection, the latter can be applied on-site. Decentralized sanitation systems are increasingly deployed globally and can provide wastewater reuse, energy and nutrient recovery. New sanitation concepts that separate blackwater (BW—toilet wastewater containing feces, urine, toilet paper and flush water) and greywater (GW—shower/washing wastewater) at the source for treatment on-site conserve organic and inorganic compounds in a smaller volume, thus facilitating recovery [5].

In decentralized sanitation systems, the BW tank is a rich nutrient environment containing 82% of the total nitrogen and 68% of the total phosphorus present in domestic wastewater [6]. This environment also features several microbial communities that play an important role in wastewater treatment [7]. However, despite the development of this technology over the last 100 years, the associated microbial composition and functions remain unclear [7]. In BW tanks, microorganisms such as the anammox group are responsible for the biological and catalytic processes involved in nitrogen removal (anaerobic ammonium oxidation) and ammonia-oxidizing Archaea [8], while bacteria from the *Ureibacillus* genus can degrade organic matter [9,10]. Based on 16S rRNA gene analyses, these processes are associated with different microbes (nitrite oxidation bacteria) that express functional genes (copper-containing nitrite reductase, nitrous oxide reductase, sulfate reductase) enabling the removal of toxins, contaminants and some nutrients [7,11]. In addition, several studies have reported that decentralized systems efficiently reduce pathogens, including bacteria (*Salmonella* and *E. coli*) [12], protozoans (*Giardia* spp.) [13], helminths (*Ascaris lumbricoides*) [14], hookworms (*Strongyloides stercoralis*) and trophozoites (*Trichomonas* and *Enterobius vermicularis*) [12]. Therefore, adequate wastewater treatment systems that produce safe treated wastewater for reuse and resources, whether centralized or decentralized, are crucial for further recovery, reuse and recycling.

**Figure 1:**
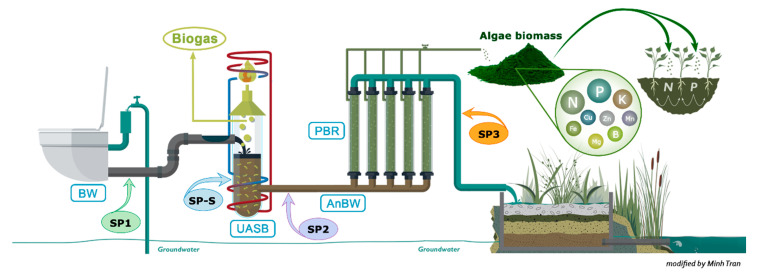
On-site black water treatment scheme at NIOO-KNAW used for the evaluation of the microbial communities in different treatment modules: blackwater (SP1), sludge from the UASB reactor (SP-S), anaerobic digested blackwater (SP2) and effluent from the tubular PBR (SP3).

The Netherlands Institute of Ecology (NIOO-KNAW) in The Netherlands has implemented such a new decentralized sanitation system in its approximately 250-person office/laboratory building. The BW is vacuum-collected using only 1 L of ground water per flush and then treated in a UASB (upflow anaerobic sludge blanket) reactor, where the carbon is converted into biogas, a renewable energy source. Anaerobic treatment of concentrated BW is a proven technology for treating high-strength wastewater [5]. The remaining effluent, known as anaerobically treated BW (AnBW), contains the major part of the nutrients and is treated in a microalgae pilot tubular photobioreactor (PBR) (Figure 1). After harvesting the microalgal biomass, the effluent is polished in a constructed wetland and finally discharged into surface water. By implementing a biological wastewater treatment strategy mimicking natural processes, we strive to achieve an efficient and robust process for wastewater reuse, carbon and nutrient recovery and a healthy final effluent that is safe to discharge. However, the microbiome composition in this system is unknown. Therefore, in this study, we assessed the dynamics of the microbial community structure and functions in this on-site BW digestion system by monitoring the influent (BW), sludge from the UASB reactor, anaerobic digested BW and finally the effluent of the UASB reactor and microalgae PBR. We hypothesize that the diversity of microbial community composition and function decrease along the different modules of the BW bioreactor due to changes in substrate composition.

## 2. Materials and Methods 

### 2.1. Blackwater Samples

BW was collected for the experiments from vacuum toilets (1 L of water/flush) at the NIOO-KNAW building (Figure 1) in Wageningen, the Netherlands (SP1). The BW consists only of feces, urine, toilet paper and approximately one liter of ground water. Anaerobic treatment of BW has proved to be the core technology for nutrient and energy recovery [6,15]. At NIOO, a UASB reactor operating at 35 °C is installed to treat the BW (UASB). The reactor has a volume of 893 L and is designed based on a hydraulic residence time (HRT) of 8.7 days, flow rate of 102.64 L d^−1^ and upflow velocity of 1.3 cm h^−1^. The diameter and height of the UASB reactor are 0.66 m and 2.75 m, respectively, with 5 taps installed every 0.46 m from the bottom to monitor the sludge bed (SP-S—first tap from the bottom). The effluent of the UASB reactor was treated in a pilot-scale tubular PBR inoculated with the microalgal species *Chlorella sorokiniana* (PBR) in continuous mode, with an HRT of 5 days (influent flow of 42.2 L d^−1^ and dilution rate of 0.2 d^−1^). The tubular PBR consists of two parallel systems that are connected to ensure homogeneous content. Each system consists of 25 m of horizontal tubes and two vertical air lift columns. At the top of the air lift columns, a cylindrical box ensures complete mixing of the tubular PBR’s content. The inner diameter of the horizontal tubes is 5.6 cm, and the inner diameter of the air lift columns is 12 cm. The horizontal tubes, airlift columns and top box have volumes of 140 L, 63 L, and 8 L, respectively, resulting in a total volume of approximately 211 L. The PBR content was homogenously mixed by air bubbling enriched with 10% CO_2_ at a flow of 10 L min^−1^. Temperature, pH and light intensity in the PBR were measured every minute and logged with NIOO logger V1.0 custom-made software. The pH was automatically controlled at 6.7 ± 0.1 by acid (2M HCl) and base (2M NaOH) addition as needed. The light intensity was calculated as the average of the values (180 μmol·s^−1^ m^−2^) obtained from the three sensors (located in three places: on both sides of the reactor and on top of the cylindrical box for every minute of every day during the daylight period.

Wastewater samples were collected from the BW (SP1), sludge from the UASB reactor (SP-S), anaerobic digested BW (SP2), and effluent from the tubular PBR (SP3) three times (t = 0, t = 1 and t = 2) with one week between each sampling time (Figure 1). Collected samples were homogenized and sub-samples were taken to perform chemical and microbial analysis. The sub-samples for microbial community analysis were immediately frozen with liquid nitrogen at the sampling moment and stored until DNA extraction. Temperature, pH and dissolved oxygen were measured during sampling with field meters.

### 2.2. Chemical Analysis

Total nitrogen (TN), total phosphorus (TP), total chemical oxygen demand (CODtotal), total suspended solids (TSS), volatile suspended solids (VSS), dissolved oxygen (DO) and alkalinity were all measured according to standard methods [16] at the chemical laboratory of the Netherlands Institute of Ecology, Wageningen, The Netherlands.

### 2.3. DNA Extraction

Samples were collected from four points of the sanitation system as described above and illustrated in Figure 1: SP1, SP-S, SP2 and SP3. For each sample, three biological replicates were obtained. The total DNA of each sample replicate was extracted from 250 mg of material using the PowerSoil^®®^ DNA Isolation Kit (MO BIO Laboratories, Inc., Carlsbad, CA, USA) according to the manufacturer’s instructions. The extracted DNA was quantified by a spectrophotometer (NanoDrop™ 2000, Thermo Fisher Scientific, MA, Wilmington, Delaware, USA) and visualized by 1.0% agarose gel electrophoresis and ethidium bromide staining. The total DNA was used for metagenome shotgun sequencing. Library preparation and Illumina HiSeq XTen sequencing were performed at BGI Genomics (Beijing, China). The resulting sequences were deposited in the European Nucleotide Archive (ENA; https://www.ebi.ac.uk/ena) under accession number PRJEB19684.

### 2.4. DNA Shotgun Metagenome Analyses

The sequence data were processed using EBI pipeline version 3 (https://www.ebi.ac.uk/metagenomics/pipelines/3.0) for classification into different functional categories: Biological Process, Cellular Component and Molecular Function (Supplementary Table S1). Then, the classified function sequences were classified according to taxonomy (Phylum, Class, Order, Family and Genus) using Kaiju http://kaiju.binf.ku.dk [17]. The potential functions and taxonomy of the microbial communities in the different treatments were analyzed as described in the Statistical analysis section.

### 2.5. Statistical Analysis

To compare the taxonomic and functional microbial structures and determine their correlations with chemical factors among the treatments, we performed canonical correspondence analysis (CCA). For this, the matrices were initially analyzed using detrended correspondence analysis (DCA) to evaluate the gradient size of the species distribution, which indicated a non-linear distributed data (length of gradient >4), revealing that the best-fit mathematical model for the data was CCA. Forward selection (FS) and Monte Carlo permutation tests were applied with 1000 random permutations to verify the significance of the physicochemical properties for the microbial community. To test whether the sample categories harbored significantly different microbial community structures, we performed permutational multivariate analysis of variance (PERMANOVA) [18]. Alpha diversity was calculated from a matrix of richness at the species level using Shannon’s index. CCA plots were generated using Canoco 4.5 software (Biometrics, Wageningen, The Netherlands), and PERMANOVA and alpha diversity indexes were calculated with the software PAST 3 [19]. To visualize differences in microbial community composition among the treatments, we generated taxonomic and functional matrices as input for the software Statistical Analysis of Metagenome Profile (STAMP) [20]. The comparison was based on *p*-values calculated using the two-sided Welch’s t-test, and correction was performed using the Benjamini-Hochberg false discovery rate [21]. For visualization, heatmaps were constructed based on the z-score-transformed phylum/function abundances to improve the normality and homogeneity of the variances. 

In addition, network analyses were performed to assess the dynamics of the interactions among the microbial communities in each module: SP1, SP-S, SP2, and SP3. Non-random co-occurrence analyses were performed using SparCC, a tool capable of estimating correlation values from compositional data [22]. We calculated SparCC correlations between microbial taxa at the genus level based on the metagenome taxonomic affiliation. For each network analysis, *p*-values were obtained by 99 permutations of random selections of the data table, subjected to the same analytical pipeline. SparCC correlations with a magnitude >0.7 or <−0.7 and statistical significance (*p* < 0.01) were included in the network analyses. The nodes in the reconstructed networks represent OTUs at the genus level, whereas the edges (that is, connections) correspond to strong and significant correlations between nodes. The topology of the network was calculated based on a set of measures, including the numbers of nodes and edges, modularity, number of communities, average path length, network diameter, average degree and clustering coefficient ([23,24]. Co-occurrence analyses were carried out using the Python module ‘SparCC’ and network visualization, and properties measurements were calculated with the interactive platform Gephi [25].

## 3. Results

### 3.1. Physicochemical Composition of the Blackwater Anaerobic Digestion System

Table 1 shows the characteristics of the BW (SP1), sludge from the UASB reactor (SP-S), anaerobic digested blackwater (SP2) and effluent from the tubular PBR (SP3) used in this study. The sewage temperature varied between 18 and 22 °C during the entire study. The total chemical oxygen (CODtotal), total nitrogen (TN), and total phosphorus (TP) varied between 5.2 and 17, 1.42 and 2.53, and 0.07 and 0.20 g L^−1^, respectively. The maximum removal of TN and TP in the UASB reactor was 44% and 53%, respectively. The CODtotal in the effluent of the tubular PBR decreased due to growth of the microalgae. During the experimental period, DO was low in SP1, SP-S and SP2, indicating anaerobic conditions. By contrast, due to photosynthetic oxygen evolution by the microalgae, an increase in the DO concentration was observed in the culture medium in SP3.

### 3.2. Microbial Community Structure

**Figure 2:**
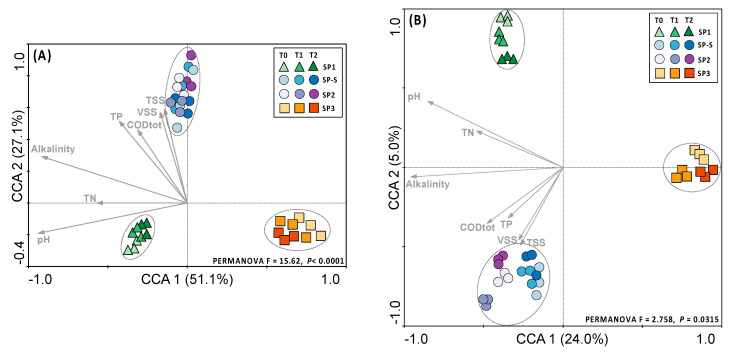
Canonical correspondence analysis (CCA) of the (**A**) taxonomic and (**B**) functional profiles and correlation with the physicochemical characteristics of on-site blackwater treatment. The arrows indicate the correlations between the physicochemical parameters and microbial profiles. Significant clusters (PERMANOVA, *p* < 0.05) are indicated by dashed lines in the graph.

To visualize the differences in community structure among the treatments, taxonomic and functional abundance matrices were used for canonical correlation analysis (CCA) (Figure 2). For taxonomy, the samples were grouped according to the treatment module (PERMANOVA F = 15.62, *p* < 0.0001), with no differences between sampling times (Figure 2A). For the functional profile, the same pattern of grouping was observed (PERMANOVA F = 2.758, *p* = 0.0315), with no differences between sampling times (Figure 2B). For both the taxonomy and functional profiles, the samples clustered in three main clusters: one for SP1, one for SP3 and one for SP2 together with SP-S (Figure 2A,B). The taxonomic and functional microbial communities assembly in SP-S and SP2 were driven by total solid suspended (TSS) and volatile solids suspended (VSS). For S3 taxonomic and functional microbial communities assembly were driven by low alkalinity condition. 

### 3.3. Microbial Community Diversity and Composition

**Figure 3:**
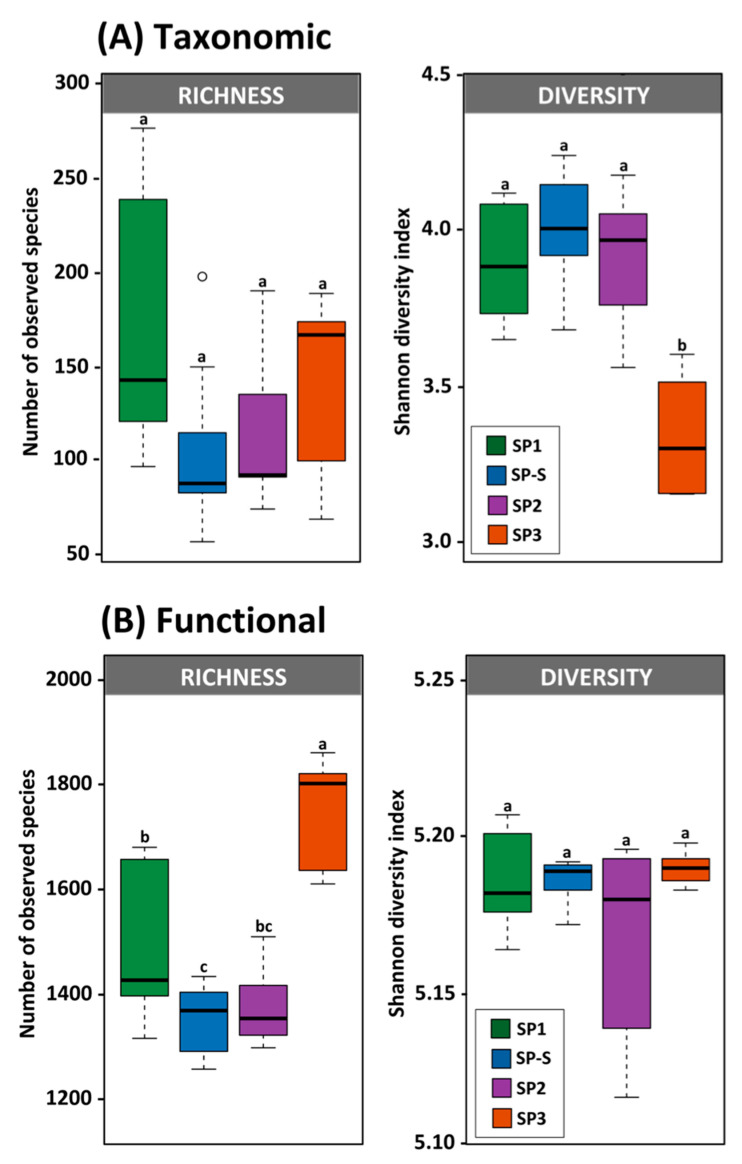
Diversity measurements of (**A**) taxonomic and (**B**) functional profiles based on DNA shotgun metagenome analysis. Different lowercase letters refer to significant differences between treatments based on Tukey’s HSD test (*p* < 0.05).

**Figure 4:**
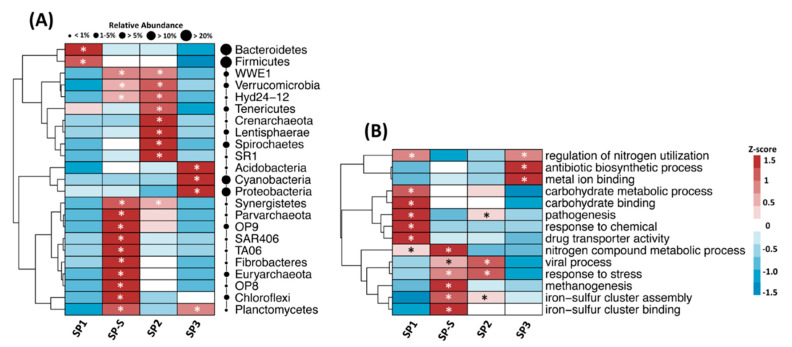
Heatmaps showing the differential abundance of (**A**) bacterial phyla and (**B**) functional categories in the on-site blackwater treatment system. The color key relates the heatmap colors to the standard score (z-score), i.e., the deviation from the row mean in units of standard deviation above or below the mean. Asterisks indicate significantly different group abundances based on the two-sided Welch’s t-test with Benjamini-Hochberg false discovery rate correction (*p* < 0.05). The circles are proportional to the relative abundance of each phylum in all samples. Only phyla with significant differences between treatment modules are included in the figure.

Approximately 48,000 sequences of acceptable quality were generated and used for downstream analysis. The species richness did not differ significantly between the treatment modules, but there is a decrease trend from SP1 to SP3, considered these two treatments, an abrupt decrease from SP1 to SP-S and then an increase for SP2 and SP3 (Figure 3A). By contrast, a decrease in diversity was observed in SP3 (Figure 3A). Comparing the effect of sampling time within each treatment module revealed increased diversity from times T0 to T2 (Supplementary Figure S1). The majority of the 16S rRNA gene sequences were affiliated with bacteria (65% of all sequences), followed by 1% with archaea. The remaining 34% of the sequences could not be affiliated and were removed from further analysis. Interestingly, the proportions of bacterial and archaeal sequences differed among the treatment modules, with higher proportions of archaea in SP2 and SP-S (USAB) should be expected (Supplementary Figure S2). The phylum Bacteroidetes dominated the samples, with an average of 30% of all sequences, followed by Firmicutes (27.6%), Proteobacteria (15%), Cyanobacteria (11.74%), and Spirochaetes (3.7%). Of the 51 total phyla affiliated with our sequences, 23 presented significant variation in abundance among the treatment modules (Figure 4A). SP1 hosted higher abundances of Bacteroidetes and Firmicutes, while SP3 presented higher abundances of Acidobacteria, Cyanobacteria, and Proteobacteria compared with the other treatment modules. SP2 and SP-S exhibited the most distinct environments, with 17 microbial phyla with higher abundances compared with SP1 and SP3, most notably Verrucomicrobia, Synergistetes, and two archaeal phyla. At a lower taxonomic level, the three most abundant genera were *Bacteroides*, *Prevotella*, and *Faecalibacterium* in SP1; *Bacteroides*, *Sphaerochaeta*, and Treponema in SP2; *Candidatus Cloacamonas*, *Clostridium*, and *Methanosaeta* in SP-S; and *Leptolyngbya*, *Acutodesmus*, and *Bacteroides* in SP3. In addition, the nitrifiers *Nitrosomonadaceae* and *Nitrobacter* were abundant only in SP3 (Supplementary Figure S3) what might be related to the increase of nitrate.

### 3.4. Microbial Functional Profile

The functional profiles of different treatment modules were annotated using Gene Ontology database and compared using STAMP. The functional diversity did not differ among the treatment modules, but the richness of functions was higher in SP3 (Figure 3B). Sampling time did not affect the overall functional diversity and richness (Supplementary Figure S4).

In general, functions related to biological processes decreased from SP1 to SP3, functions related to cellular components were lower in SP1 and increased in the other treatments, and molecular functions were higher in SP1 and SP3 (Supplementary Figure S5). A heatmap of selected functions that differed among the treatments is shown in Figure 4B (for a complete list of functions, see Supplementary Table S1). The most prevalent core functions in SP1 were related to metabolism of carbohydrates, response to chemicals and drugs, and nitrogen. The core functions in SP2 and SP-S were related to response to stress, viral processes and iron-sulfur cluster assembly. Methanogenesis and iron-sulfur cluster binding-related functions were most abundant in SP-S. SP3 presented high abundances of functions related to antibiotic biosynthetic processes and metal ion binding. SP1 and SP3 had regulation of nitrogen utilization as a core function. Interestingly, the abundance of sequences related to ‘pathogenesis’ drastically decreased from SP1 to SP-S and from SP2 to SP3.

### 3.5. Co-Occurrence Network Analysis

**Figure 5:**
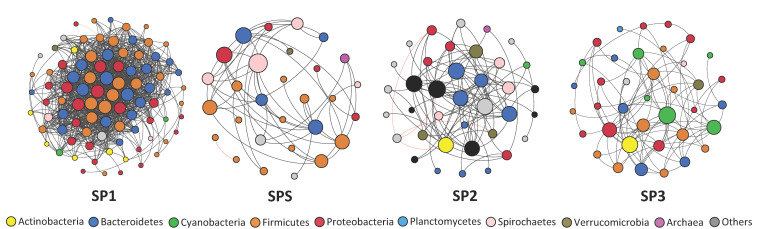
Network co-occurrence analysis of microbial communities from the on-site blackwater treatment system. A connection indicates a SparCC correlation with a magnitude of >0.7 (positive correlation—black edges) or <−0.7 (negative correlation—red edges) and statistical significance (*p* < 0.01). Each node represents a different bacterial genus, and the size of the node is proportional to the number of connections (degree). Each node is labelled at the phylum level.

To explore the complexity and dynamics of the microbial communities in each treatment module, we conducted a co-occurrence network analysis by calculating SparCC correlations between microbial taxa at the genus level based on the 16S rRNA data. We further calculated the topological properties of the obtained networks to identify differences between the treatment modules. The network analyses showed that community complexity drastically decreased from SP1 to SP-S, increased from SP-S to SP2 and decreased from SP2 to SP3 (Figure 5 and Supplementary Table S2). SP1 was the most complex (89 nodes; 1081 edges; 7 community hubs; average degree of 24.29), followed by SP2 (37 nodes; 95 edges; 7 community hubs; av. degree of 5.13), SP3 (38 nodes; 86 edges; 6 hubs; av. degree of 4.52), and finally SP-S, the least complex (29 nodes; 51 edges; 6 hubs; av. degree of 3.51).

## 4. Discussion

The average COD_total_, TN, and TP in the concentrated BW were 17.0, 2.53 and 0.20 g L^−1^, respectively. The concentration of COD_total_ in the BW in the present study is higher than that reported by Zeeman et al. [5] and De Graaff et al. [15] for BW collected from vacuum toilets, but the concentrations of TN and TP are similar. The pH of the UASB reactor effluent was 7.70, within the recommended range for methanogenesis according to Chernicharo [26]. Alkalinity was higher in the effluent of the UASB reactor than in the influent, which is desirable because high concentrations of volatile acids can be buffered without causing a substantial drop in pH [26]. Appropriate total alkalinity can maintain the buffering capacity of the reactor at favorable levels [27]. The average COD_total_ removal in the UASB reactor was 60.6%, lower than the value reported by De Graaff et al. [15] due to some periods of instability in the influent flow of the reactor. The average removal of TN and TP in the UASB reactor was 30.4 and 30.0%, respectively; the majority of TN and TP remained in the liquid phase for subsequent removal/recovery in the tubular PBR. The TSS concentration in the sludge of the UASB reactor was 45.8 g L^−1^, which is classified as dense and flocculent according to Chernicharo (2017) [26]. The COD_total_ in the effluent of the tubular PBR decreased because of microalgal growth. The removal/recovery of TN and TP was 44.0% and 65.0%, respectively, from the influent to the tubular PBR. 

Our results showed that the BW module increased microbial richness and diversity. From a microbial perspective, BW offers a wider niche for the UASB reactor, which likely promoted increased microbial community diversity via organic matter concentration. However, along the different modules of the bioreactor, the abundance of DNA sequences decreased dramatically. The network analysis showed a decrease in the microbial community complexity from the beginning SP1 to SPS, concomitant to a reduced diversity in final step of the bioreactor (SP3). Such a decrease is reasonable and reflects both changes in substrate composition and wash conditions. In the initial stage, the BW promoted increases in Bacteroidetes, Firmicutes, and Proteobacteria, which accounted for more than 60% of all DNA sequences affiliated to bacteria. Previous studies have reported the presence and high abundance of Bacteroidetes in human feces and water environments [28,29,30]. Bacteroidetes play an important role in acidification in UASB sludge [28,30]. Moreover, Bacteroidetes are a group of proteolytic bacteria with a robust capacity to decompose proteins, amino acids, and high-molecular-weight compounds in water environments, explaining the dominance of this phylum in the initial phase [31,32].

The high abundance and strong shifts in the phyla Bacteroidetes, Firmicutes, and Proteobacteria dominated the composition of the common microbiome in the BW module [33]. This is because Bacteroidetes and Firmicutes are capable of degrade various types of cellulose, mainly from toilet paper [30]. In the initial phase at BW there is a large amount of cellulose biomass which contributed to select cellulose-degrading bacteria. The high abundance of cellulose-degrading bacteria found in our study play an important role on fiber decomposition. The fiber decomposition compound can accumulate and hazardous impact the other steps such as sludge production and biogas production during the anaerobic processes [34]. Moreover, the predominance of Firmicutes in the SP1 tanks indicate a dependence on molecules produced by other groups of microorganisms once these syntrophic bacteria are capable of degrading cellulosic compounds and producing volatile fatty acids (VFAs) and H_2_, which are then consumed by hydrogenotrophic methanogens in metabolic reactions [35]. The third most abundant phylum, Proteobacteria, predominated in the SP3 tanks. This phylum comprises sulfate-reducing groups of bacteria that, like Firmicutes, are syntrophic and depends on symbioses (associated with methanogens) [36]. 

We also found significantly higher abundances of Acidobacteria and Cyanobacteria in the SP3 tanks. Members of the phylum Cyanobacteria are able to thrive in several critical conditions in bioreactor tanks. According to Stal [37], Cyanobacteria oxidize sulfides under anoxic conditions and perform anoxygenic photosynthesis utilizing a hydrogen sulfide-like electron donor. In a study of biofilms containing Cyanobacteria in scum from a bioreactor, [38] observed spatial proximity between Cyanobacteria and hydrogen sulfide bacteria. 

The microbial community also showed several different functions along the bioreactor. The SP1 tank promoted functions related to the metabolism of carbohydrates, chemicals, drugs, and nitrogen. Human waste sewage is composed mainly of high concentrations of nitrogen and carbohydrates [39,40]. Organic nitrogen is the major nitrogen form in human feces. Therefore, the transformation of organic nitrogen to its inorganic forms (ammonia/ammonium) requires an association with bacterial groups such as Bacteroidetes and Firmicutes [41,42]. Those groups likely transformed the nitrogen in the bioreactor system since they were the most abundant groups in the first stage of water decontamination. Bacteroidetes and Firmicutes also degrade carbohydrates in the human gut [43,44], their original habitat before transitioning to the bioreactor. In SP1, we also identified other functions related to cellular components such as stimulus, molecule transport, and pathogenesis. All these functions are likely associated with the high diversity of fecal components, which stimulates cell activity in the initial phase of the bioreactor.

In SP-S, we detected nitrogen metabolic processes resulting from initial nitrogen transformation. We also detected several other functions related to the exchange of electron acceptors and anaerobic transformation of waste. The synthrophic groups *Synergistetes*, *Parvachaeota*, and *Chloroflexi* play important roles in anaerobic processes [11,45,46]. According to [45], the predominance of these methanogenic groups indicates a syntrophic relationship with other bacterial groups. In the present study we also found high correlation with methanogenesis process in the SP-S. This function is directly related to the methanogenic degradation of toilet paper [47]. The water insoluble cellulose from toilet paper are a significant fraction of sewage what carbohydrate decomposition is very [48]. We here found that the partial degraded cellulose by Bacteroidetes and Firmicutes from SP1 activated the methanogenesis process from methanogenic *Chloroflexi* and *Synergistetes* at the SP-S which converted cellulose in methane. 

In SP3, we found a high abundance of functions related to regulation of nitrogen, antibiotic biosynthetic and metal ion binding. Those functions are related to microalgal activity, which has shown efficient performance in decontaminating water [49,50,51]. In addition, these same microalgae have been shown to be used as rich nutrient in barley production [52]. Our system of wastewater treatment also decreased microbial functions related to pathogenesis, and the analyses confirmed that the microalgae-containing SP3 reduced bacterial richness and diversity. In SP3, the oxygen and pH value variation induced by microalgae photosynthesis reduces coliforms and other pathogenic bacteria. Microalgal CO_2_ uptake can cause the pH to rise to 10–11, this increase is beneficial for the disinfection of pathogens. However, the pH was automatically controlled at 6.7 ± 0.1, but there was an increase of DO concentration in SP3 as a result of photosynthetic activity contributes with pathogenic bacteria removal. UV light penetration and temperature also could contribute with the pathogenic bacteria removal. Natural antibiotics and other metabolites produced by microalgae and adhesion to the cell surface of microalgae aid in the reduction of pathogenic organisms [53,54]. The algal system could reduce pathogens to a non-detectable level within five days of processing, which may be attributed to the combination of low pH and other operating conditions such as high temperature, what findings are consistent with those of Delanka-Pedige [55]. The findings of this study contribute to understand the dynamics of the microbial community structure and functions in this on-site BW digestion system either at treatment scale or environmental scale. However, future studies on active microbiome (i.e., metatranscriptome) in BW digestion system located in different places should be considered. 

## 5. Conclusions

In summary, the new pilot sanitation system decreases microbial richness and diversity followed by a decrease in microbial functional complexity and co-occurrence along the different modules of the bioreactor. The sanitation system fosters microbial groups and functions that enable an efficient and robust process for carbon and nutrient recovery and apparently reduce potential pathogenic groups, resulting in a healthy final effluent that is much safer for discharge.

## Figures and Tables

**Table 1:** Characteristics of the blackwater system in different treatment modules: blackwater (SP1), sludge from the UASB reactor (SP-S), anaerobic digested blackwater (SP2) and effluent from the tubular PBR (SP3).

Points of Sampling	COD_total_ (g L^−1^)	TN (g L^−1^)	TP (g L^−1^)	Alkalinity (g L^−1^)	pH	TSS (g L^−1^)	VSS (g L^−1^)	T (°C)	DO Mg L^−1^
	Average ± standard variation								
SP1	17.0 ± 4.7	2.53 ± 1.89	0.20 ± 0.05	4.85 ± 1.14	8.40 ± 0.30	8.2 ± 3.1	7.5 ± 3.1	18.0 ± 1.8	0.58 ± 1.02
SP-S	43.7 ± 3.4	3.85 ± 1.49	0.46 ± 0.08	5.19 ± 0.10	7.60 ± 0.40	45.8 ± 0.9	36.0 ± 1.2	21.6 ± 6.3	0.36 ± 0.57
SP2	6.7 ± 2.8	1.76 ± 1.26	0.14 ± 0.02	5.19 ± 0.10	7.70 ± 0.10	4.8 ± 2.4	4.6 ± 2.2	23.3 ± 9.3	0.63 ± 0.71
SP3	5.2 ± 2.6	1.42 ± 1.21	0.07 ± 0.01	0.44 ± 0.21	6.60 ± 0.40	2.8 ± 0.4	2.5 ± 0.1	22.0 ± 2.6	1.10 ± 0.62

COD_total_ = total chemical oxygen demand; TSS = total solids suspended, VSS = volatile solids suspended.

## Data Availability

The sequences were deposited in the European Nucleotide Archive (ENA; https://www.ebi.ac.uk/ena) under accession number PRJEB19684.

## Supplementary Materials

Supplementary materials can be found at https://www.mdpi.com/2076-2607/9/1/75/s1. Figure S1. Taxonomic diversity measurements comparing the effect of sampling time (T0, T1 and T2) between modules (treatments) of the on-site blackwater treatment system. Different lowercase letters indicate significant differences between treatments based on Tukey’s HSD test (*p* < 0.05); Figure S2. General proportions of sequences affiliated with bacteria and archaea in different modules (treatments) of the on-site blackwater treatment system. Different lowercase letters indicate significant differences between treatments based on Tukey’s HSD test (*p* < 0.05); Figure S3. Total number of sequences affiliated with nitrifiers in the on-site blackwater treatment system; Figure S4. Functional diversity measurements comparing the effect of sampling time (T0, T1 and T2) between modules (treatments) of the on-site blackwater treatment system. Different lowercase letters indicate significant differences between treatments based on Tukey’s HSD test (*p* < 0.05); Figure S5. General proportions of sequences affiliated with functional categories. Different lowercase letters indicate significant differences between treatments based on Tukey’s HSD test (*p* < 0.05); Table S1. Proportion of sequences (%) affiliated to GeneOnthology Database. *p*-values indicate significant differences based on two-sided Welch’s t-test corrected by using Benjamini-Hochberg FDR; Table S2. Correlations and topological properties of the microbiome networks.
