# Supplementary material for: On-Site Blackwater Treatment Fosters Microbial Groups and Functions to Efficiently and Robustly Recover Carbon and Nutrients
Source: Microorganisms. 2020 Dec 30;9(1):75. doi: 10.3390/microorganisms9010075 (PMC7824102; doi:10.3390/microorganisms9010075)
Supplement: Supplementary file 1 [file microorganisms-09-00075-s001.pdf]

# On-Site Blackwater Treatment Fosters Microbial Groups and Functions to Efficiently and Robustly Recover Carbon and Nutrients

**Eiko E. Kuramae [1,2,*], Mauricio R. Dimitrov [1], Gustavo H. R. da Silva [3], Adriano R. Lucheta [1], Lucas W. Mendes [1], Ronildson L. Luz [1], Louise E. M. Vet [4] and Tania V. Fernandes [5]**

[1] Department of Microbial Ecology, Netherlands Institute of Ecology (NIOO-KNAW), Droevendaalsesteeg 10, 6708 PB Wageningen, The Netherlands; mau_dimitrov@hotmail.com (M.R.D.); arlucheta@gmail.com (A.R.L.); l.mendes@nioo.knaw.nl (L.W.M.); luzrnd@gmail.com (R.L.L.)
[2] Ecology and Biodiversity, Institute of Environmental Biology, Utrecht University, Padualaan 8, 3584 CH Utrecht, The Netherlands
[3] Department of Environmental and Civil Engineering, São Paulo State University (UNESP), Bauru, 17033-360, Brazil; gustavo.ribeiro@unesp.br
[4] Department of Terrestrial Ecology, Netherlands Institute of Ecology (NIOO-KNAW), Droevendaalsesteeg 10, 6708 PB Wageningen, The Netherlands; l.vet@nioo.knaw.nl
[5] Department of Aquatic Ecology, Netherlands Institute of Ecology (NIOO-KNAW), Droevendaalsesteeg 10, 6708 PB Wageningen, The Netherlands; T.Fernandes@nioo.knaw.nl
* Correspondence: E.Kuramae@nioo.knaw.nl

**Supplementary Material**

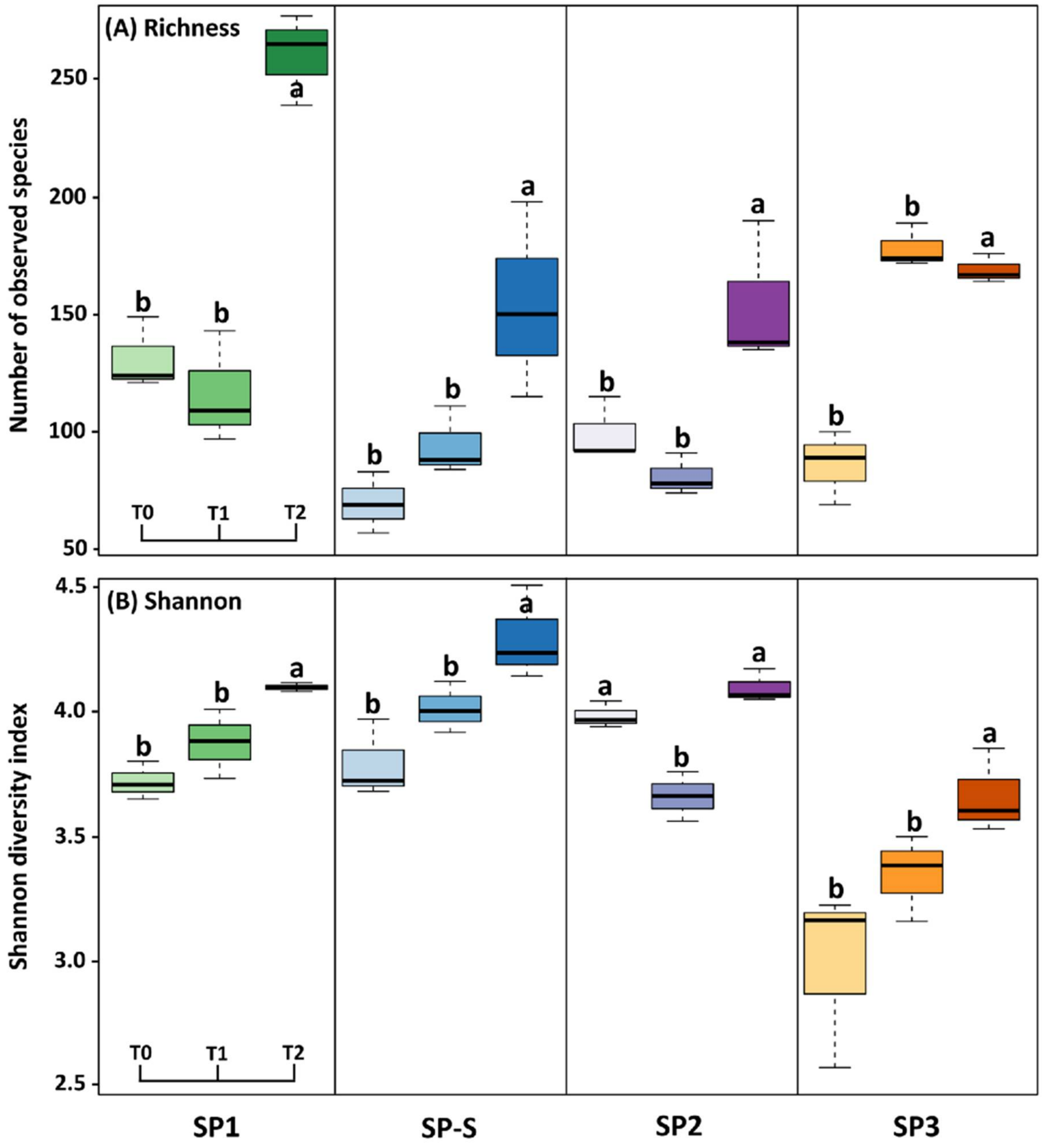


**Figure S1.** Taxonomic diversity measurements comparing the effect of sampling time (T0, T1 and T2) between modules (treatments) of the on-site blackwater treatment system. Different lowercase letters indicate significant differences between treatments based on Tukey's HSD test ($p < 0.05$).

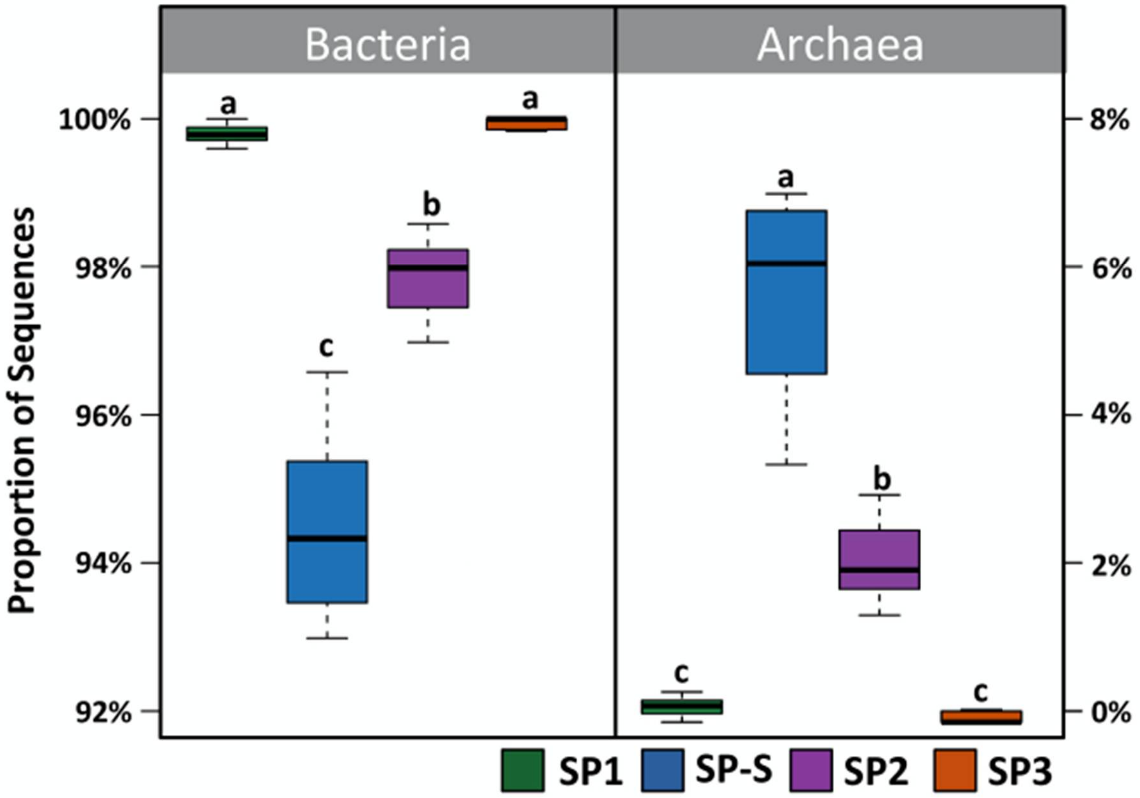


**Figure S2.** General proportions of sequences affiliated with bacteria and archaea in different modules (treatments) of the on-site blackwater treatment system. Different lowercase letters indicate significant differences between treatments based on Tukey's HSD test ($p < 0.05$).

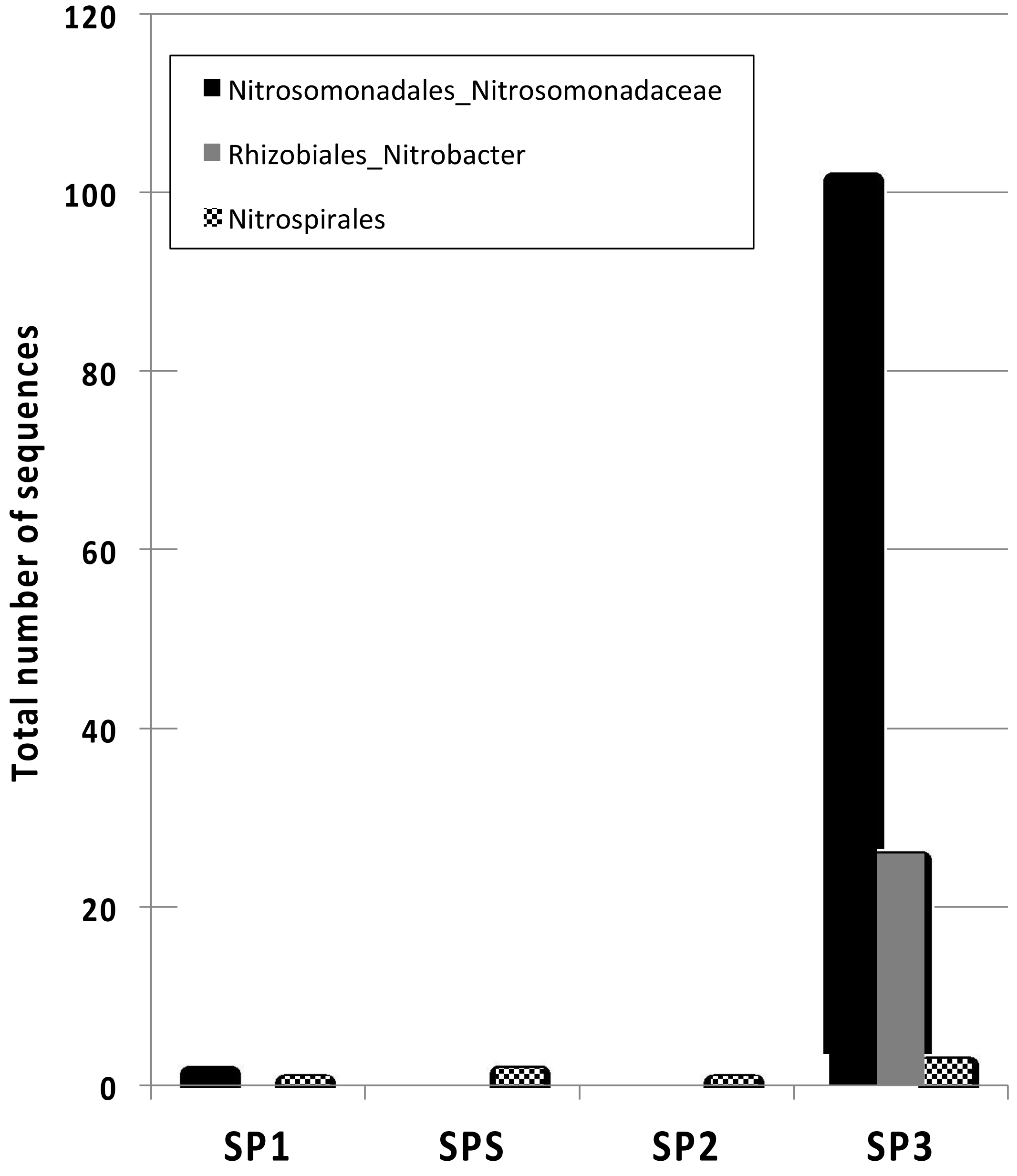


**Figure S3.** Total number of sequences affiliated with nitrifiers in the on-site blackwater treatment system.

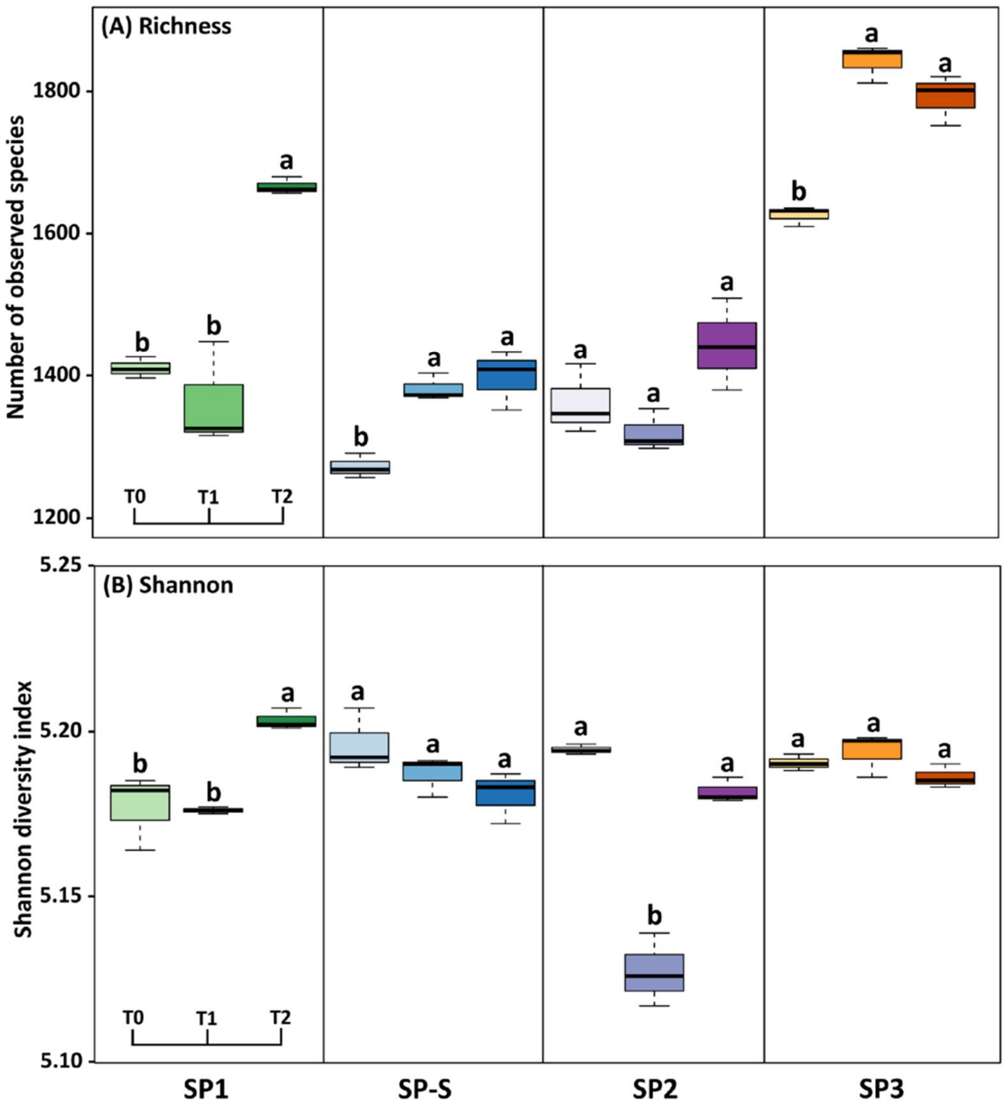


**Figure S4.** Functional diversity measurements comparing the effect of sampling time (T0, T1 and T2) between modules (treatments) of the on-site blackwater treatment system. Different lowercase letters indicate significant differences between treatments based on Tukey's HSD test ($p < 0.05$).

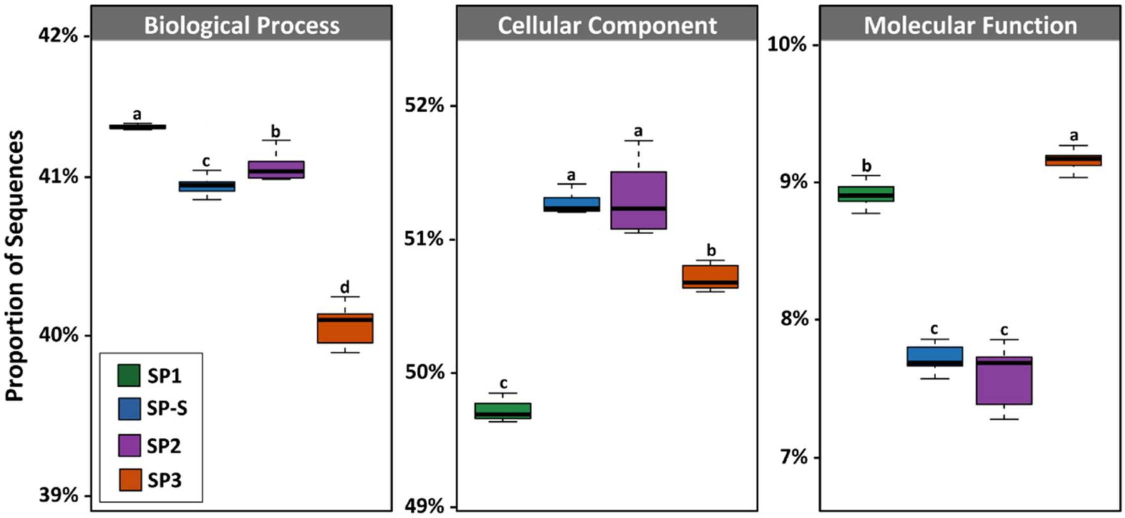


**Figure S5.** General proportions of sequences affiliated with functional categories. Different lowercase letters indicate significant differences between treatments based on Tukey's HSD test ($p < 0.05$).

**Table S1.** Proportion of sequences (%) affiliated to GeneOnthology Database. *p*-values indicate significant differences based on two-sided Welch's t-test corrected by using Benjamini-Hochberg FDR.

| Category | Description | Proportion of Sequences | | | | *p*-value |
|---|---|---|---|---|---|---|
| | | SP1 | SP2 | SP-S | SP3 | |
| Biological Process | photosynthesis | 0.001 | 0.001 | 0.002 | 0.268 | 2.73E-34 |
| | generation of precursor metab. and energy | 0.356 | 0.333 | 0.339 | 0.649 | 3.85E-34 |
| | metabolic process | 5.452 | 6.091 | 6.678 | 8.156 | 3.85E-28 |
| | carbohydrate metabolic process | 3.428 | 2.844 | 2.724 | 1.861 | 1.07E-27 |
| | phosphorelay signal transduction system | 0.564 | 0.398 | 0.471 | 0.863 | 1.21E-26 |
| | signal transduction | 0.285 | 0.244 | 0.271 | 0.515 | 1.03E-24 |
| | antibiotic biosynthetic process | 0.005 | 0.009 | 0.016 | 0.041 | 3.79E-24 |
| | sporulation | 0.037 | 0.012 | 0.008 | 0.003 | 4.07E-24 |
| | small molecule metabolic process | 4.348 | 4.443 | 4.441 | 3.863 | 2.50E-23 |
| | response to abiotic stimulus | 0.001 | 0.004 | 0.002 | 0.022 | 6.50E-23 |
| | RNA metabolic process | 1.907 | 2.148 | 1.998 | 1.385 | 4.19E-22 |
| | DNA conformation change | 0.490 | 0.466 | 0.411 | 0.276 | 6.07E-22 |
| | cytochrome complex assembly | 0.025 | 0.021 | 0.030 | 0.072 | 8.69E-22 |
| | transport | 4.918 | 3.733 | 3.935 | 4.638 | 4.98E-21 |
| | regulation of metabolic process | 1.329 | 1.015 | 1.094 | 1.347 | 1.66E-20 |
| | biosynthetic process | 4.204 | 4.295 | 4.213 | 3.765 | 2.72E-20 |
| | methanogenesis | 0.004 | 0.012 | 0.051 | 0.010 | 2.02E-19 |
| | translation | 1.996 | 2.284 | 2.094 | 1.489 | 3.36E-19 |
| | cellular component organiz. or biogenesis | 0.555 | 0.592 | 0.547 | 0.404 | 7.56E-19 |
| | response to biotic stimulus | 0.022 | 0.037 | 0.041 | 0.015 | 2.36E-18 |
| | quorum sensing | 0.022 | 0.003 | 0.002 | 0.002 | 2.83E-18 |
| | biological process | 0.304 | 0.208 | 0.211 | 0.296 | 1.70E-16 |
| | viral process | 0.027 | 0.055 | 0.049 | 0.020 | 8.33E-16 |
| | macromolecular complex assembly | 0.019 | 0.013 | 0.011 | 0.028 | 1.04E-14 |
| | response to chemical | 0.290 | 0.165 | 0.152 | 0.167 | 4.29E-14 |
| | phosphorylation | 0.725 | 0.773 | 0.830 | 0.942 | 9.34E-14 |
| | cell redox homeostasis | 0.088 | 0.092 | 0.101 | 0.137 | 1.50E-13 |
| | regulation of nitrogen utilization | 0.014 | 0.008 | 0.005 | 0.015 | 2.21E-12 |
| | DNA metabolic process | 2.994 | 3.718 | 3.075 | 2.041 | 3.48E-12 |
| | protein folding | 0.242 | 0.255 | 0.230 | 0.196 | 8.00E-12 |
| | regulation of DNA repair | 0.015 | 0.015 | 0.013 | 0.007 | 4.03E-11 |
| | cell adhesion | 0.013 | 0.017 | 0.019 | 0.029 | 1.58E-10 |
| | response to stress | 0.700 | 0.767 | 0.755 | 0.669 | 4.03E-09 |
| | proteolysis | 1.050 | 1.151 | 1.219 | 1.019 | 8.53E-08 |
| | lipid metabolic process | 0.643 | 0.661 | 0.644 | 0.708 | 4.52E-07 |
| | bioluminescence | 0.003 | 0.014 | 0.005 | 0.002 | 9.52E-07 |
| | pathogenesis | 0.016 | 0.011 | 0.006 | 0.007 | 4.53E-05 |
| | toxin biosynthetic process | 0.010 | 0.010 | 0.008 | 0.007 | 0.000156 |
| | nitrogen compound metabolic process | 3.399 | 3.359 | 3.453 | 3.358 | 0.000196 |
| | iron-sulfur cluster assembly | 0.064 | 0.070 | 0.073 | 0.068 | 0.000667 |
| | transcription, DNA-templated | 0.683 | 0.649 | 0.665 | 0.639 | 0.001417 |
| | bacterial-type flagellar cell motility | 0.067 | 0.063 | 0.071 | 0.082 | 0.001900 |
| | viral entry into host cell | 0.001 | 0.001 | 0.001 | 0.000 | 0.009792 |
| Cellular Component | membrane protein complex | 0.006 | 0.005 | 0.004 | 0.238 | 2.33E-33 |
| | Thylakoid | 0.001 | 0.001 | 0.001 | 0.229 | 1.08E-32 |
| | intrinsic to membrane | 1.558 | 1.272 | 1.371 | 1.969 | 5.57E-32 |
| | Cytoplasm | 0.869 | 0.911 | 0.883 | 0.671 | 1.27E-23 |
| | Membrane | 3.828 | 2.783 | 2.968 | 3.824 | 3.49E-23 |
| | Nucleus | 0.002 | 0.001 | 0.002 | 0.023 | 2.57E-19 |
| | extrinsic component of membrane | 0.000 | 0.000 | 0.000 | 0.009 | 2.85E-19 |
| | extrachromosomal circular DNA | 0.072 | 0.026 | 0.011 | 0.003 | 3.00E-19 |
| | beta-galactosidase complex | 0.074 | 0.031 | 0.032 | 0.004 | 3.00E-18 |
| | acetyl-CoA carboxylase complex | 0.022 | 0.015 | 0.014 | 0.035 | 3.41E-17 |
| | cell wall | 0.025 | 0.010 | 0.008 | 0.010 | 2.38E-16 |
| | Ribosome | 0.857 | 0.942 | 0.840 | 0.643 | 1.01E-14 |

| | | | | | | |
|---|---|---|---|---|---|---|
| | oxidoreductase complex | 0.018 | 0.014 | 0.015 | 0.028 | 1.41E-14 |
| | Intracellular | 0.781 | 0.808 | 0.728 | 0.632 | 5.42E-14 |
| | outer membrane | 0.128 | 0.108 | 0.109 | 0.143 | 4.56E-12 |
| | periplasmic space | 0.084 | 0.081 | 0.108 | 0.156 | 1.24E-11 |
| | molybdopterin synthase complex | 0.004 | 0.003 | 0.004 | 0.007 | 2.19E-11 |
| | proteasome core complex | 0.004 | 0.010 | 0.013 | 0.008 | 4.27E-11 |
| | proton-transp. two-sector ATPase complex | 0.112 | 0.101 | 0.115 | 0.091 | 3.21E-10 |
| | citrate lyase complex | 0.006 | 0.008 | 0.009 | 0.000 | 9.73E-10 |
| | Virion | 0.011 | 0.019 | 0.009 | 0.006 | 1.56E-09 |
| | Chromosome | 0.072 | 0.081 | 0.079 | 0.058 | 7.87E-09 |
| | extracellular region | 0.034 | 0.025 | 0.021 | 0.024 | 3.18E-08 |
| | DNA polymerase complex | 0.060 | 0.066 | 0.062 | 0.048 | 2.15E-07 |
| | bacterial type flagellum | 0.045 | 0.039 | 0.049 | 0.056 | 7.80E-06 |
| | ATP-binding cassette (ABC) transp. comp. | 0.058 | 0.069 | 0.079 | 0.066 | 8.83E-06 |
| | Microtubule | 0.000 | 0.000 | 0.001 | 0.003 | 1.33E-05 |
| | plasma membrane | 0.121 | 0.097 | 0.112 | 0.120 | 1.73E-05 |
| | cellular component | 0.013 | 0.018 | 0.016 | 0.018 | 8.98E-05 |
| | signal recognition particle | 0.020 | 0.022 | 0.021 | 0.015 | 0.00012 |
| | catalytic complex | 0.014 | 0.015 | 0.018 | 0.020 | 0.00069 |
| | phosphopyruvate hydratase complex | 0.013 | 0.013 | 0.014 | 0.010 | 0.00266 |
| | DNA helicase complex | 0.008 | 0.008 | 0.008 | 0.006 | 0.03521 |
| | Gram-negative-bacterium-type cell wall | 0.001 | 0.000 | 0.000 | 0.000 | 0.05084 |
| | riboflavin synthase complex | 0.017 | 0.016 | 0.017 | 0.015 | 0.49673 |
| **Molecular Function** | electron carrier activity | 0.123 | 0.093 | 0.135 | 0.624 | 5.92E-39 |
| | tetrapyrrole binding | 0.191 | 0.200 | 0.252 | 0.747 | 3.18E-32 |
| | oxidoreductase activity | 3.109 | 3.485 | 3.916 | 5.372 | 5.48E-31 |
| | hydrolase activity | 3.611 | 3.339 | 3.186 | 2.601 | 1.57E-30 |
| | drug transporter activity | 0.218 | 0.086 | 0.068 | 0.031 | 3.89E-30 |
| | peroxidase activity | 0.025 | 0.022 | 0.030 | 0.082 | 9.14E-28 |
| | isomerase activity | 1.446 | 1.385 | 1.302 | 0.915 | 1.41E-27 |
| | transcription factor activity | 0.688 | 0.451 | 0.432 | 0.636 | 4.86E-25 |
| | transporter activity | 2.566 | 1.974 | 2.045 | 2.705 | 6.27E-25 |
| | receptor activity | 1.264 | 0.515 | 0.584 | 1.151 | 6.52E-25 |
| | coenzyme binding | 0.918 | 1.076 | 1.144 | 1.229 | 3.09E-24 |
| | metal ion binding | 1.454 | 1.531 | 1.599 | 1.970 | 1.04E-23 |
| | signal transducer activity | 0.360 | 0.274 | 0.314 | 0.536 | 1.45E-23 |
| | nucleotidyltransferase activity | 0.829 | 0.883 | 0.841 | 0.617 | 1.26E-22 |
| | protein binding | 1.245 | 1.549 | 1.690 | 2.205 | 1.92E-22 |
| | recombinase activity | 0.184 | 0.096 | 0.083 | 0.056 | 2.41E-22 |
| | ligase activity | 1.801 | 2.029 | 1.994 | 1.454 | 2.06E-21 |
| | nucleotide binding | 6.770 | 7.443 | 7.372 | 6.397 | 2.11E-21 |
| | carbohydrate binding | 0.325 | 0.223 | 0.215 | 0.137 | 4.49E-21 |
| | vitamin binding | 0.190 | 0.306 | 0.347 | 0.230 | 1.53E-20 |
| | molecular function | 0.489 | 0.423 | 0.476 | 0.641 | 4.54E-20 |
| | catalytic activity | 5.172 | 5.500 | 5.767 | 5.254 | 4.72E-20 |
| | iron-sulfur cluster binding | 0.305 | 0.408 | 0.495 | 0.389 | 1.28E-19 |
| | antioxidant activity | 0.080 | 0.084 | 0.107 | 0.156 | 8.79E-18 |
| | transcription factor binding | 0.080 | 0.120 | 0.165 | 0.074 | 9.16E-18 |
| | transferase activity | 3.370 | 3.725 | 3.786 | 3.348 | 6.82E-15 |
| | structural constituent of ribosome | 0.864 | 0.950 | 0.844 | 0.649 | 1.43E-14 |
| | amino acid binding | 0.118 | 0.119 | 0.125 | 0.081 | 1.52E-14 |
| | nucleic acid binding | 6.122 | 6.598 | 5.751 | 4.571 | 8.93E-14 |
| | ion binding | 0.011 | 0.011 | 0.009 | 0.020 | 6.58E-12 |
| | phosphatase activity | 0.104 | 0.077 | 0.085 | 0.073 | 6.18E-11 |
| | kinase activity | 0.860 | 0.878 | 0.885 | 0.982 | 3.88E-09 |
| | nucleoside-triphosphatase activity | 1.867 | 1.830 | 1.864 | 1.663 | 7.37E-09 |
| | pyridoxal phosphate binding | 0.392 | 0.429 | 0.426 | 0.481 | 1.07E-08 |
| | penicillin binding | 0.142 | 0.117 | 0.112 | 0.108 | 2.47E-08 |
| | lyase activity | 0.941 | 0.905 | 0.937 | 1.000 | 5.10E-07 |

| | | | | | | |
|---|---|---|---|---|---|---|
| | peptidase activity | 1.125 | 1.282 | 1.375 | 1.211 | 5.87E-07 |
| | transposase activity | 0.383 | 0.905 | 0.535 | 0.334 | 0.000197 |

**Table S2.** Correlations and topological properties of the microbiome networks.

| **Network properties** | **SP1** | **SP-S** | **SP2** | **SP3** |
|---|---|---|---|---|
| Number of nodes[a] | 89 | 29 | 37 | 38 |
| Number of edges[b] | 1081 | 51 | 95 | 86 |
| Positive edges[c] | 1079 | 50 | 91 | 86 |
| Negative edges[d] | 2 | 1 | 4 | 0 |
| Modularity[e] | 0.139 | 0.352 | 0.384 | 0.364 |
| Number of communities[f] | 7 | 6 | 7 | 6 |
| Network diameter[g] | 6 | 5 | 6 | 8 |
| Average path length[h] | 2.019 | 2.602 | 2.494 | 2.842 |
| Average degree[i] | 24.29 | 3.517 | 5.13 | 4.52 |
| Av. clustering coefficient[j] | 0.741 | 0.445 | 0.573 | 0.469 |

[a] Microbial taxon (at genus level) with at least one significant ($p < 0.01$) and strong (SparCC > 0.7 or <−0.7) correlation; [b] Number of connections/correlations obtained by SparCC analysis; [c] SparCC positive correlation (> 0.7 with $p < 0.01$); [d] SparCC negative correlation (<−0.7 with $p < 0.01$); [e] The capability of the nodes to form highly connected communities, that is, a structure with high density of between nodes connections (inferred by Gephi); [f] A community is defined as a group of nodes densely connected internally (Gephi); [g] The longest distance between nodes in the network, measured in number of edges (Gephi); [h] Average network distance between all pair of nodes or the average length off all edges in the network (Gephi); [i] The average number of connections per node in the network, that is, the node connectivity (Gephi); [j] How nodes are embedded in their neighborhood and the degree to which they tend to cluster together (Gephi).
